# SG-ATT: A Sequence Graph Cross-Attention Representation Architecture for Molecular Property Prediction

**DOI:** 10.3390/molecules29020492

**Published:** 2024-01-19

**Authors:** Yajie Hao, Xing Chen, Ailu Fei, Qifeng Jia, Yu Chen, Jinsong Shao, Sanjeevi Pandiyan, Li Wang

**Affiliations:** 1 School of Information Science and Technology, Nantong University, Nantong 226001, China; 2110310030@stmail.ntu.edu.cn (Y.H.); 2230310036@stmail.ntu.edu.cn (X.C.); 2230310037@stmail.ntu.edu.cn (A.F.); 2230310054@stmail.ntu.edu.cn (Q.J.); 2110310019@stmail.ntu.edu.cn (Y.C.); sylershao@gmail.com (J.S.); gpsanjeevi@gmail.com (S.P.); 2 Research Center for Intelligent Information Technology, Nantong University, Nantong 226001, China

**Keywords:** molecular graph, knowledge graph, cross-attention, molecular property prediction

## Abstract

Existing formats based on the simplified molecular input line entry system (SMILES) encoding and molecular graph structure are designed to encode the complete semantic and structural information of molecules. However, the physicochemical properties of molecules are complex, and a single encoding of molecular features from SMILES sequences or molecular graph structures cannot adequately represent molecular information. Aiming to address this problem, this study proposes a sequence graph cross-attention (SG-ATT) representation architecture for a molecular property prediction model to efficiently use domain knowledge to enhance molecular graph feature encoding and combine the features of molecular SMILES sequences. The SG-ATT fuses the two-dimensional molecular features so that the current model input molecular information contains molecular structure information and semantic information. The SG-ATT was tested on nine molecular property prediction tasks. Among them, the biggest SG-ATT model performance improvement was 4.5% on the BACE dataset, and the average model performance improvement was 1.83% on the full dataset. Additionally, specific model interpretability studies were conducted to showcase the performance of the SG-ATT model on different datasets. In-depth analysis was provided through case studies of in vitro validation. Finally, network tools for molecular property prediction were developed for the use of researchers.

## 1. Introduction

Currently, well-designed molecular representations, such as molecular fingerprints or molecular descriptors, can achieve good results for specific tasks. However, most molecular characterizations based on feature engineering are optimized for specific tasks and lack generality [1,2,3]. With the development of deep neural networks, various molecular representation models have freed researchers from complex and time-consuming feature engineering [4,5]. Molecular characterization learning has also officially entered the era of deep learning, e.g., in compound property prediction, drug screening of small molecules, drug interactions, and more foreseeable applications [6,7,8]. 

Molecular property prediction methods based on molecular graphs and SMILES sequence-based molecular properties have rapidly developed [9,10,11]. To address the problem of inadequate representation of molecular features in a single dimension [12], Pang et al. proposed a multidimensional molecular feature encoding model in 2021. They used the molecular SMILES sequence encoding as input and obtained the 1D and 2D feature vectors of molecules using the transformer model and message-passing neural networks (MPNN) model, respectively [13,14,15]. In addition, basic chemical domain knowledge is an important prior knowledge in molecular information, and work has been implemented to utilize domain prior chemical knowledge for multimodal learning [16]. Combining the above ideas, Fang et al. proposed the knowledge-enhanced contrastive learning (KCL) framework to construct a chemical element knowledge graph (CKG) based on the periodic table of elements to expand the molecular graph by expanding relationships between elements and their basic chemical properties on the original graph. The expanded molecular graph contains the topology of the molecule and basic chemical domain knowledge of the element [17]. Hasebe’s work proposes a knowledge-embedded message-passing neural network [18]. This network can combine molecular properties and knowledge annotations on molecular graphs as supervision to learn molecular properties and structures. Incorporating knowledge embedding into the model for multimodal learning of molecular attributes and manually annotated knowledge makes the graph neural networks (GNNs) more generalizable and physically consistent. The debut of ChatGPT has attracted great attention from the natural language processing (NLP) community [19]. Large language models have proved their ability to perform various NLP tasks. With the success of language processing models based on transformer architecture in many fields, related researchers have also attempted to introduce it and pre-train with fine-tuning models for molecular characterization [20]. The topological information of the molecule is expressed by aggregating information according to the presence or absence of edges between atoms when aggregating information after self-attention has been computed during pre-training.

In current molecular property prediction tasks, molecular representations are usually represented using only molecular graphs or SMILES sequence encoding. However, neither type of input can express sufficiently rich molecular semantic and structural information on its own. Because SMILES sequences alone encode a complex molecular spatial structure compressed into a single linear sequence, they cannot encode the spatial structure of the molecule itself. The molecular graph-based approach heavily depends on the amount of data, and it performs worse than the molecular descriptor-based approach when the dataset is small. In addition, GNNs are prone to over-smoothing problems, so the number of GNN layers is usually only two to four, which limits their feature extraction capability. In addition, the method overemphasizes the importance of GNNs for structure perception and ignores the generalization of the graph structure.

Obtaining models with sufficient prediction accuracy requires large datasets for pre-training. It is difficult to achieve training goals using small datasets. One solution is to use transfer learning, which can improve the prediction accuracy for tasks with small datasets [21]. However, to apply transfer learning, another dataset with molecular properties related to the target properties must be available. Therefore, to address the universality of prediction models across different datasets, multimodal learning using prior knowledge of physics and chemistry has improved the prediction model performance by incorporating knowledge into deep learning. For example, existing molecular representation models usually encode atoms in a molecular graph as individuals who can interact only in the presence of chemical bonds but do not consider the correlations between atoms, which qualitatively exist between unconnected atoms in the molecular graph. If a knowledge graph is constructed based on the relationships between elements using domain knowledge, then the original molecular graph can be enhanced through knowledge-guided expansion. This expansion helps establish connections between atoms that share common properties but are not directly connected via chemical bonds.

Combining these two points, the multidimensional feature encoding that combines 1D and 2D features complements the structural information of molecules implicitly in SMILES and reduces the requirement of relevant molecular representation models for GNN to extract implicit chemical information, which can further improve the accuracy of prediction tasks. In addition, if domain knowledge is applied to the prediction of molecular attributes, it is helpful for the model to capture the interaction between atoms and the characteristics of the molecular substructure.

To solve these problems, multidimensional molecular feature encoding is explored and combined with a molecular knowledge graph (MKG), and the SG-ATT model is proposed. Thus, the SG-ATT can learn molecular features from 1D semantic and 2D topological structure perspectives. In the process of learning molecular graph features, the proposed MKG incorporates features at both the atom and functional group levels, thereby enhancing the representation capability of molecular graphs. After feature encoding, a cross-attention method is used to extract molecular features adaptively based on different weighted molecular features, and finally, a composite molecular representation is obtained.

The main contributions of SG-ATT are as follows:

(1) Fusing 1D and 2D molecular features encoding to learn molecular representations from a multidimensional view.
(2) Considering the generalization ability of the model for small datasets, the molecular graph is enhanced using MKG guidance, which allows the model to learn richer molecular information.
(3) Adopting a cross-attention mechanism to improve the learning ability of the representation of molecular.

## 2. Results

### 2.1. Dataset and Setup

To benchmark the SG-ATT model performance, nine widely used molecular property-related datasets were selected from the MoleculeNet dataset, covering physical chemistry (ESOL, FreeSolv), Quantum mechanics (QM7), biophysics (BACE, HIV), and physiology (BBBP, Tox21, ToxCast, SIDER, and ClinTox) [22]. The number of tasks in these datasets is 1 to 617, and the size of the datasets is 642 to 41,127. Table A1 in Appendix C summarizes the details of the datasets. 

### 2.2. Benchmarking Methods

The SG-ATT model was compared with the following baseline methods to test its performance on downstream tasks. The graph convolutional network (GCN) and model Weave are two types of graph convolutional methods [23,24]. The Message-Passing Neural Network (MPNN) considers the edge features and strengthens the message interactions between bonds and atoms during message passing [15]. Directed Message Passing Neural Networks (DMPNNs) and Communicative Message-Passing Neural Networks (CMPNNs) are improvements to MPNN [25,26]. The Communicative Message Passing Transformer (CoMPT) considers edge features and enhances the message interaction between bonds and atoms in the message-passing process [27]. Fusion network of molecular fingerprints and molecular graphs for molecular property prediction (FP-GNN) [28].

(1) GCN is a convolutional method that focuses on learning the relationship with the nearest neighbor node [23].
(2) Weave transformed feature vectors using pair features with distant atoms in addition to atom features focusing only on atoms [24]. MPNN utilizes features from nodes and edges and then summarizes them into a framework [15].
(3) DMPNN treated the molecular graph as an edge-oriented directed structure, avoiding information redundancy during iterations [25].
(4) CMPNN improves molecular embedding by enhancing message interaction between nodes and edges through communication kernel [26].
(5) CoMPT invokes a communicative message-passing paradigm based on Transformer [27].
(6) FP-GNN combines and simultaneously learns information from molecular graphs and fingerprints for molecular property prediction [28].

### 2.3. Experimental Setup

Using the Adam optimizer with an initial learning rate of 0.0001 and a batch size set to 64, these configurations are implemented through PyTorch (version 1.12.0) and the Deep Graph Library [29,30]. For the graph encoder, the dimension size of the feature vector is set to 128. In the sequence encoder, the number of attention heads in the transformer is set to 8, and the final feature length is set to 128. To prevent overfitting, an early stopping mechanism is employed to ensure SG-ATT runs for a maximum of 100 epochs. Hyperparameter selection is performed using a random search approach. Additionally, for each experimental run, the average of results from 10 random seeds is taken. All code development is conducted on a GPU (NVIDIA GeForce 3080ti, NVIDIA, Santa Clara, CA, USA) utilizing an Ubuntu server.

### 2.4. Experimental Results and Analysis

In this section, we tested whether the proposed SG-ATT model approach outperformed the benchmark approach. Table 1 provides the following observations. Firstly, SG-ATT consistently achieved the best performance across all regression datasets, the best results on three classification datasets, and the second-best result on one dataset. The significant overall dataset performance demonstrates the effectiveness of SG-ATT in molecular property prediction tasks. Furthermore, SG-ATT exhibited a relative improvement of 2.9% over the state-of-the-art (SOTA) baseline in the small dataset FreeSolv, which comprises only 642 labeled molecules. This result confirms the advantage of SG-ATT in small datasets for several reasons. Firstly, the model leverages functional group-level knowledge, amplifying its capacity to capture distinctive features of hydrophilic functional groups such as hydroxyl (-OH) and amino (-NH_2_), as well as hydrophobic functional groups like alkyl (-CH_3_). These functional groups often exert a crucial influence on a molecule’s solubility in various solvents. Secondly, the SG-ATT model seamlessly integrates multidimensional features and chemical element knowledge, empowering it not only to glean more nuanced molecular representations from limited datasets but also to enhance its generalization prowess across a spectrum of datasets.

**Figure 1:**
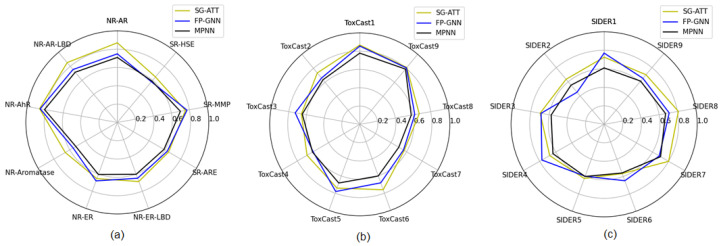
The test results of attribute tasks on multitask datasets. (**a**) represents the testing results on the Tox21 dataset; (**b**) represents the testing results on the ToxCast dataset; (**c**) represents the testing results on the SIDER dataset.

To further validate the model’s performance, we treated each task on the Tox21, ToxCast, and SIDER datasets as a separate attribute and then compared ROC-AUC among tasks within each dataset. Figure 1 illustrates the comparison of ROC-AUC for nine attribute tasks on each dataset using different models. Our model achieved state-of-the-art performance in 20 out of the total 27 attribute tasks.

### 2.5. Ablation Experiments

The ablation study was conducted to verify the importance of multidimensional features in the SG-ATT model on model performance. The experiments were repeated five times on each encoder to obtain stable results. Table 2 shows the experimental results of SG-ATT.

The bold cells indicate the best results obtained through SG-ATT, and the underlined cells indicate the second-best results obtained via SG-ATT. w/o Graph indicates the removal of the graph feature module in the SG-ATT model, and w/o Sequence indicates the removal of the sequence feature module in the SG-ATT model. The experimental results in Table 2 show that the exclusion of either component (Transformer and AMPNN) can easily degrade the model performance. Excluding the AMPNN module causes more obvious model performance degradation, which proves the effectiveness of AMPNN in improving SG-ATT performance. The SG-ATT experiments obtain the worst results if both components are simultaneously excluded. The ablation experiments confirm that the SG-ATT model can learn richer molecular representations and improve the prediction performance of downstream tasks.

### 2.6. Exploration of Model Interpretability

**Figure 2:**
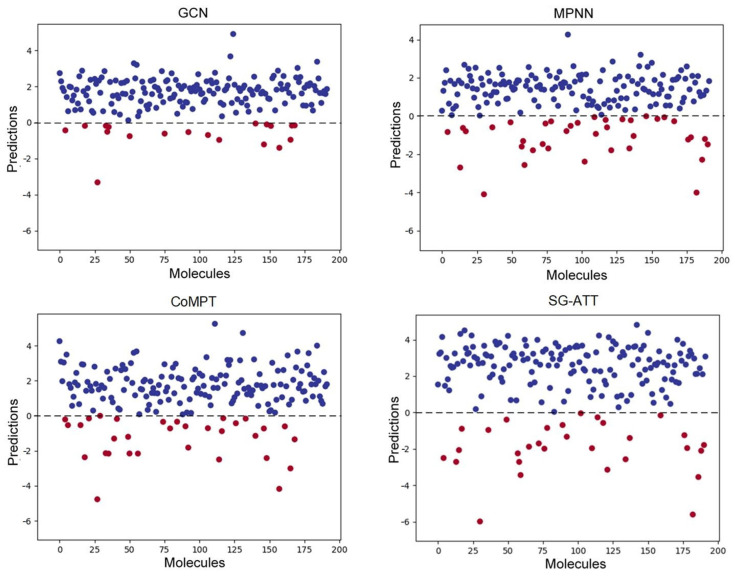
BBBP dataset model test results.

Figure 2 depicts the prediction results of the four models on the BBBP test set, with red dots representing instances predicted with a label of 0 and blue dots representing instances predicted with a label of 1. When predictions are entirely accurate, there are a total of 42 instances with a label of 0 in the BBBP test set, represented by red dots. However, in the prediction plot of GCN, only 18 instances are predicted to be 0, whereas in SG-ATT, 30 instances are predicted to be 0. This highlights that the SG-ATT model, through the fusion of multi-dimensional features and prior knowledge, can capture rich features of different molecular types. Consequently, compared to other baseline models, the SG-ATT model yields more accurate prediction results.

**Figure 3:**
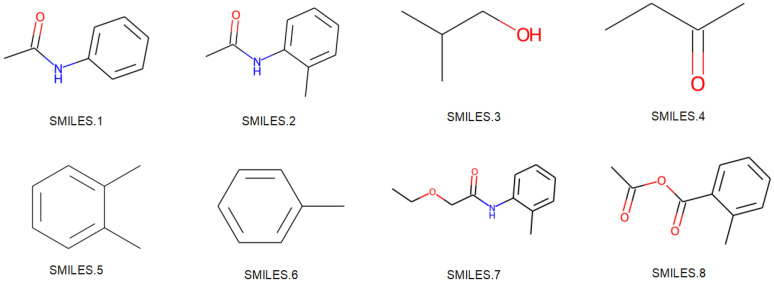
Molecules with different scaffolds.

**Figure 4:**
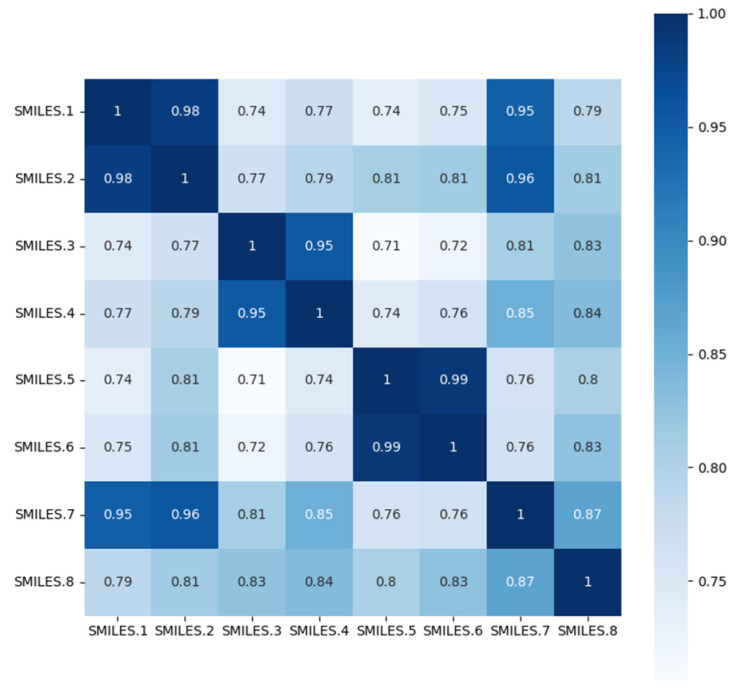
Similarity heatmap of molecules with different scaffolds.

To validate whether the molecular embeddings learned through the SG-ATT model align with real-world knowledge, experiments were conducted for the blood–brain barrier permeability task using randomly selected eight molecules with different scaffolds. Different molecules are depicted in Figure 3, where distinct molecular scaffolds exhibit both similar and dissimilar cases. As shown in Figure 4, the trained model was employed to obtain embedding vectors for the eight molecules. The cosine similarity between different vectors was computed to assess the features learned through the model concerning molecular characteristics for various scaffolds. The specific SMILES representations for each molecular graph are provided in Appendix D.

**Figure 5:**
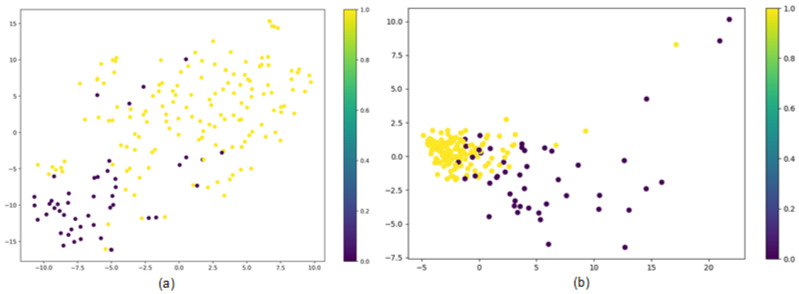
t-SNE feature dimension reduction. (**a**) represents the results of the MPNN model trained on the BBBP dataset, while (**b**) represents the results of the SG-ATT model trained on the same dataset.

T-distributed random neighbor embedding (t-SNE) is a machine learning algorithm used for data dimensionality reduction, visualizing high-dimensional data, and providing an intuitive understanding of the data distribution. To further investigate the effectiveness of SG-ATT in learning molecular features, the dimensionality of the embedding vectors learned through the SG-ATT model was reduced to 2D using the t-SNE visualization method. As shown in Figure 5, it can be observed that the molecular embedding vectors obtained through SG-ATT can clearly distinguish active molecules from inactive ones with higher classification accuracy. Additionally, the embeddings of different active molecules exhibit a more clustered pattern. Therefore, SG-ATT can learn differentiated feature representations among different active molecules and capture similar features among molecules of the same class, which is crucial for its outstanding performance in molecular property prediction tasks.

### 2.7. Case Analysis

Chronic hepatitis B caused by hepatitis B virus (HBV) is a kind of serious disease. HBV inhibitory drugs are developed from a variety of compounds, and to further explore whether anti-HBV compounds are potentially promising for research, it is necessary to validate their anti-HBV capacity (IC_50_) and HepG2 hepatocytotoxicity (CC_50_). We used compounds screened with the fields of target name and target tissue from the CHEMBL database as training data and set the HBV (IC_50_) concentration and hepatotoxicity concentration (CC_50_) thresholds at 20 μM (the smaller the IC_50_ value; the more specific the performance of the antibody) and 100 μM (higher CC_50_ concentration indicates lower toxicity), respectively, to convert the compound activity data against the target into binary data for prediction [31]. To validate the reliability of the SG-ATT model, we also used in vitro validation to test the model on IC_50_ and CC_50_ to predict the properties of the compounds. Throughout the in vitro validation, we tested the activity data of 100 compounds for IC_50_ and CC_50_. Table 3 shows the final test results of CC_50_ and IC_50_ on CHEMBL data and in vitro validation data.

We counted the model prediction results and listed the top 10 compounds ranked using μM prediction size. The first represents the largest μM, and the 10th represents the smallest μM. Table 4 shows the IC_50_ prediction results and in vitro validation results obtained through the SG-ATT model for compounds with specified thresholds, and Table 5 shows the CC_50_ prediction results and in vitro validation results of the model for compounds with specified thresholds. The tests of IC_50_ and CC_50_ for compounds not present in the training data show that the model can predict different physicochemical properties of compounds as well as generalizability to different datasets.

### 2.8. Web Server Implementation

**Figure 6:**
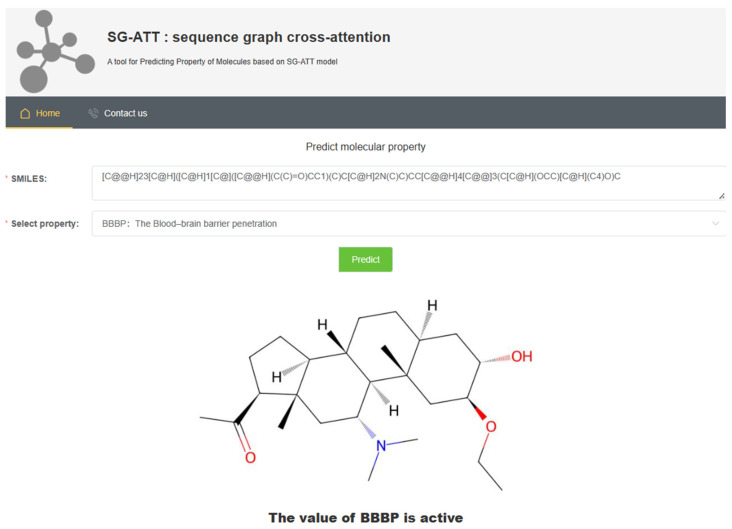
Screen capture of the web tool driven by SG-ATT.

To facilitate researchers in implementing the proposed SG-ATT model, an easy-to-use web server was built on http://vectorspaceai.cn/SG-ATT (accessed on 12 June 2023). The server interface is shown in Figure 6. To obtain the desired results, the user only needs to upload the SMILES string of the compound of interest and select the desired predicted molecular properties. Finally, the service returns the molecular graph of the corresponding compound along with the predicted results of the desired property.

## 3. Materials and Methods

Molecular property prediction can be considered as classification tasks and regression tasks to determine whether a molecule has certain activities. In the SG-ATT approach, a molecule is represented by a sequence of SMILES, which consists of a series of chemical atoms and chemical bonds. The sequence of SMILES is denoted as $S_{l}=\left\{s_{1},s_{2},\cdots s_{i}\right\}$, where *i* is the length of $S_{l}$.

**Figure 7:**
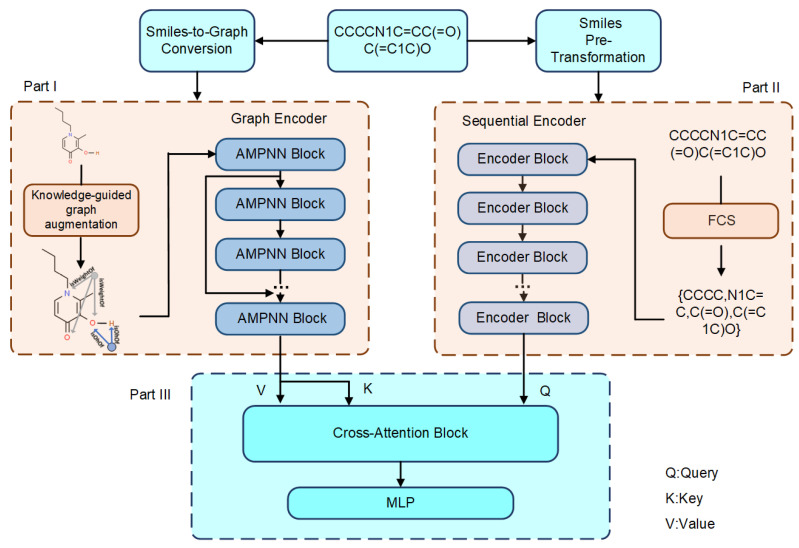
SG-ATT model framework.

In this section, the general framework of the SG-ATT model is first introduced; then, the specific methods of each module are described. The structure of the SG-ATT model is shown in Figure 7. The input to SG-ATT is a sequence of one-dimensional molecular SMILES. In part I, the SMILES sequence is converted into a molecular graph using the RDKit toolkit; then, the original molecular graph is enhanced through MKG and used as the input to the graph encoder. For the graph encoder, atomic feature vectors of the molecules are generated by combining feature attention through the atom-aware message-passing neural network (AMPNN). In part II, the SMILES sequence is directly encoded using the frequent consecutive subsequence (FCS) algorithm to generate sequence data as input to the sequence encoder [32]. The sequence encoder utilizes the transformer architecture to encode the sequence data directly and generate a feature vector that represents the one-dimensional sequence of molecules. In part III, 2D molecular graph feature vectors and 1D sequence feature vectors are fed into a cross-attention block. The feature mixing part generates a high-dimensional vector on which a decoder is implemented for downstream molecular property prediction tasks.

### 3.1. Feature Extraction

SMILES is converted to undirected graphs using the RDKit toolkit, denoted as $G=\left\{V,E\right\}$ [33], where *V* is a set of n nodes, and E is a series of edges. $e_{ij}$ is the edge $\left(i,\;j\right)$, representing the initial feature. Let *h(j)* be the node hiding state and $h\left(e_{ij}\right)$ be the edge hiding state. Learning the graph encodes, ${f=G\to R}^{d}$, which encodes the input graph into a vector representation with no labels.

The SG-ATT model applies a data-driven sequence pattern mining algorithm called the FCS algorithm. This algorithm can progressively decompose the SMILES sequence of a molecule into smaller subsequences. Molecular properties are often determined using specific substructures, and FCS decomposes molecular SMILES sequences into medium-sized substructures that are more likely to provide clear indications. The specific representation is shown in Equation (1):

(1) $$FCS\left(s_{l}\right)=D_{i}=\left\{d_{1},d_{2}\ldots d_{q}|d_{k}\in C\right\}$$

where *C* is the FCS algorithm vocabulary, s is the molecular SMILES code, and *D* is the *s* FCS subsequence.

### 3.2. Sequence Encoding: Transformer

The Transformer encoder relies on an attention mechanism to compute contextual features, which is significantly different from the Recurrent Neural Network and Convolutional Neural Networks [34,35]. The Transformer encoder is suitable for encoding sequential information and has been widely used in NLP. The multiheaded attention mechanism enables the Transformer encoder to learn the features of different subsequences in a sequence. When processing sequence information, the Transformer encoder can associate different positions of the sequence to obtain embeddings that contain contextual information.

The FCS-encoded sequence is input to the Transformer encoder for sequence encoding to generate a feature vector that contains 1D sequence-structure information $T_{transformer}$. The specific process is shown in Equations (2) and (3).

(2) $$D=FCS\left(s\right)$$

and

(3) $$T_{1}=Transformer\left(D\right).$$

### 3.3. Knowledge Graph Enhancement

**Figure 8:**
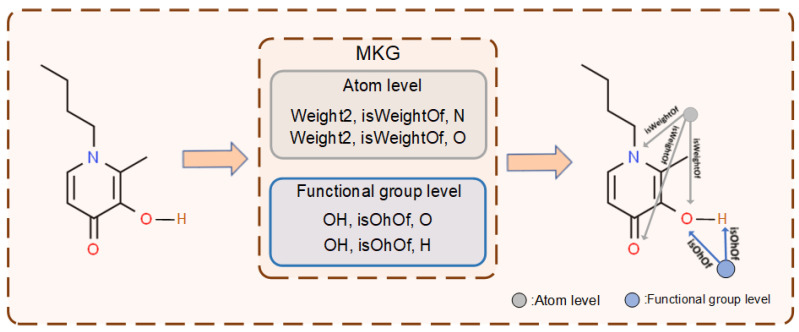
Molecular knowledge graph enhancement process.

Previous work has been conducted to construct knowledge graphs (KGs) from public chemical databases and scientific literature to extract associations between chemicals and disease or drug pairs [36,37,38]. Considering previous research, it is known that microscopic connections exist even between atoms in a molecule that are directly connected through chemical bonds [17]. Based on this approach, MKG creates triples based on the chemical elements in the periodic table of the chemical elements and their properties, with each triple containing five properties: periodicity, metallicity, group, radius, and weight. Moreover, functional groups in a molecule often play a crucial role in determining its properties. Recognizing this importance, MKG has incorporated information on commonly found functional groups in molecules. When it is found that a certain substructure exists in the molecule, a virtual node connecting the atoms is established among the substructure according to the guidance of MKG to capture the information of the substructure. Specifically, as shown in Figure 8, a connection is established between an atom in a molecule extracted in the MKG, and a 1-hop neighbor property based on that atom, and a triple is added as an edge. Since some element attributes are continuous, it is difficult for MKG to model their associations. Therefore, continuous element attributes are sampled into discrete grouping labels (e.g., radius group 1 and radius group 2). For example, at the atomic level, a node “Weight2” and an edge from “Weight2 (divide into the second group based on weight.)” to “O (oxygen)” are added to the original molecular graph based on the triple (Weight2, isweightOf, O). Similarly, at the functional group level, two edges from “OH (oxhydryl)” to “O (oxygen)” and “H (Hydrogenium)” are added, respectively. Each edge between a property and an atom is oriented from the former to the latter, while the edges between atoms are bidirectional. Similarly, when a molecular fragment is found in the molecule, a connection is established in the substructure through the obtained atomic number. Then, an augmented molecular graph is obtained, which preserves the original molecular structure and introduces virtual nodes to capture long-distance atomic interactions and molecular substructure information. The augmented molecular graph $G^{\prime }$ considers the microscopic correlations between atoms, containing richer and more complex information.

### 3.4. Molecular Graph Encoding: AMPNN

**Figure A1:**
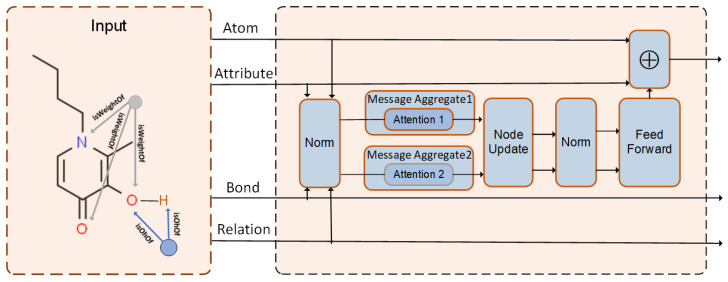
Architecture of AMPNN.

With the inclusion of domain knowledge extracted from MKG in the augmented molecular graph, KMPNN is proposed to enhance the feature encoding of the molecular graph [17]. However, considering the inclusion of different types of molecular information in the augmented molecular graph, AMPNN extends the feature aggregation method of KMPNN. After the message-passing process, it assigns different feature aggregation methods to various types of messages. By employing distinct aggregation methods, the model can better capture the features of each message type, enhancing the richness of information. Figure A1 in Appendix A describes the detailed flow of AMPNN. The key idea behind AMPNN is to use two types of message-passing channels to encode different types of neighboring nodes. During the message-passing process, a feature fusion method based on the attention mechanism is used to better incorporate the correlations between features into the model. The input to the encoder is an augmented molecular graph $G^{\prime }=\left\{V,E\right\}$. Let $N_{i}$ denote the set of neighbors of node *i*, and $X_{j}$ is the initial feature of node *j*. Then, *K* rounds of messaging are applied to all nodes. Heterogeneous message passing is enabled using two $M_{t}$ functions, where $M_{t1}$ is applied to the attributes in the neighbors, and $M_{t2}$ is applied to the neighbors that represent atoms.

In addition, the message-passing process is extended via a self-attentive mechanism, where the attention coefficients are calculated using Equation (4) and normalized through the Softmax function to make the coefficients easily comparable among different nodes [39].

(4) $$\alpha_{uv}=\frac{exp(LeakyReLU(a^{T}[Wh_{u}||Wh_{v}])}{\sum_{K\in N_{v}}exp(LeakyReLU(a^{T}[Wh_{u}||Wh_{v}])},$$

where ${\left(\cdot \right)}^{T}$ denotes the transposition, and ||  denotes the crosstalk operation. $a^{T}$ is a parameter vector with a dimension of $R^{2F}$, where *F* is the dimension of the node feature vector and serves as a parameter for the feedforward neural network. Based on this attention coefficient, we can derive Equation (5):

(5) $${h_{i}}^{\prime }=\sigma (\alpha_{ij}W_{1}{h_{e(ij)}}^{K-1}),$$

where $\alpha_{ij}$ represents the attention coefficient between attributes, and $W_{1}$ is the weight matrix for relationships. The message passing function is represented using Equation (6):

(6) $$M_{t1}={h_{i}}^{\prime }\cdot {h_{i}}^{k-1}.$$

Due to the varying importance of messages passed from different neighboring atoms to the central atom, neighboring atoms adhere to a common process with distinct parameters, as depicted in Equations (7)–(9).

(7) $$\beta_{ij}=\frac{exp(LeakyReLU(b^{T}[Wh_{i}||Wh_{j}])}{\sum_{K\in N_{i}}exp(LeakyReLU(b^{T}[Wh_{i}||Wh_{j}])},$$

(8) $${h_{i}}^{\prime }=\sigma (\beta_{ij}W_{2}{h_{e(ij)}}^{K-1}),$$

and

(9) $$M_{t2}={h_{i}}^{\prime }\cdot {h_{i}}^{k-1}$$

where $\beta_{ij}$ represents the attention coefficient between atoms, and $W_{2}$ is the weight matrix for bonds. During the message-passing process, aggregation collects messages from neighboring edges, as shown in Equations (10) and (11).

(10) $$e_{ij}^{K-1}=M_{t}({h_{e(ij)}}^{K-1}\cdot {h_{i}}^{k-1})$$

and

(11) $$e_{i}^{K}=Aggregate(e_{ij}^{K-1}),$$

where aggregation types include summation and averaging. Furthermore, the node’s hidden state is updated using the GRU function, depicted in Equation (12).

(12) $$h^{K}\left(v\right)=\;\mathrm{GRU}(h^{K-1}\left(e_{uv}\right),{\;e}_{i}^{K}(v)),$$

where GRU is a gated recurrent neural network [40]. After *K* iterations, a readout operator (13) is applied to obtain a graph-level representation of the molecule.

(13) $$T_{kmpnn}^{i}=Set2Set\left(h^{K}\left(v\right)|v\in G_{i}^{\prime }\right),$$

where Set2Set is specifically designed to operate on sets and is more expressive than simply summing the final node states [41].

### 3.5. Cross-Attention Feature Fusion

The first of the feature vectors is passed into the feature blending section, which blends the features from the 1D sequence feature encoder and 2D graphical feature encoder. This information is combined through a cross-attention mechanism (14):

(14) $$Attention\left(Q,K,V\right)=Softmax(\frac{QK^{T}}{\sqrt{C/d}})\cdot V,$$

where *Q* is created from the output of the sequence encoder, and *K* and *V* are generated from the output of the graph encoder using the projection functions $f=w^{T}x+b$ (where *w* and *b* are weight and bias, respectively). *C* and *d* are the embedding dimensions and the number of heads, respectively.

When predicting molecular properties, the multidimensional features of molecules are encoded into a feature decoder. This feature decoder then produces a final predicted label that indicates the molecular properties. Using a decoder consisting of a three-layer feedforward neural network, a strong correlation can be established between input features and output results.

### 3.6. Loss Function

To build a molecular property prediction model, a two-channel multidimensional molecular feature encoder is constructed. The transformer is applied to the sequence feature encoder, and the enhanced graph is input to the graph feature encoder, AMPNN. The multidimensional feature decoder decodes all embedding vectors from the embedding space. For the classification task, the output of the decoder is a label of 0 or 1, where 1 indicates that the predicted molecule has a certain activity, and 0 indicates that no predicted molecule has a certain activity. For the regression task, the output of the decoder is a logistic value. The SG-ATT model is optimized using the binary cross-entropy loss function; for the regression task, the RMSE loss function is used, where *y* denotes the true label and *x* denotes the model prediction, as shown in Equations (15) and (16).

(15) $${Loss}_{classification}=-\sum \left[yln\left(x\right)+\left(1-y\right)\ln\left(1-x\right)\right]$$

and

(16) $${\;\;Loss}_{regression}=\sqrt{\frac{1}{m}\sum_{i=1}^{m}{\left(y-x\right)}^{2}}.$$

Backpropagation is propagated from the output layer to the previous layer. With this end-to-end approach, the model is trained with all trainable parameters. The results show that end-to-end training can greatly improve the performance of the model because all trainable parameters accept the gradient of the loss function. In this study, the losses are propagated through a two-channel multidimensional molecular feature encoder and a multidimensional feature decoder.

## 4. Conclusions

Combining MKG-guided molecular graph enhancement, connections are established between atoms with similar attributes as well as between atoms within the same substructure. This knowledge-enhanced molecular graph is believed to better learn the information transfer between nodes to supplement the shortcomings of insufficient long-distance information transmission and tends to overlook substructure features in the molecular graph. In addition, many existing works focus on the feature learning of molecular substructures, which are obtained by directly slicing the molecular substructure to replace the original molecular graph or by fusing the substructure with the original graph for features. We aim to supplement important substructure information in molecules from a sequence perspective by slicing the molecular SMILES sequence with the FCS algorithm.

This study introduces the SG-ATT model, a multidimensional molecular feature attention encoding based on knowledge graphs designed for predicting molecular properties. By integrating multidimensional features and knowledge graph characteristics, the SG-ATT model’s ability to capture molecular information in limited datasets has been enhanced, thereby improving its generalization capabilities across diverse datasets. Additionally, the incorporation of functional group information allows the SG-ATT model to learn structural features that have a significant impact on molecular properties, consequently enhancing the model’s predictive accuracy. Through comparative experiments, visual studies, and case analyses, we demonstrated the improved performance of the SG-ATT model. However, existing research does have certain limitations that require attention. On the one hand, current Molecular Knowledge Graphs (MKGs) primarily integrate atomic and functional group features, leaving ample room for further expansion. On the other hand, the one-dimensional sequence encoding module holds the potential for more significant optimization, such as incorporating atom distance matrices to reinforce self-attention mechanisms. In our upcoming work, we are committed to addressing these limitations and refining the model accordingly.

## Figures and Tables

**Table 1:** Comparison of data from molecular attribute prediction models.

Metric	ROC-AUC				RMSE		MAE
Dataset	BBBP	BACE	HIV	ClinxTox	ESOL	FreeSolv	QM7
Weave [2016]	0.837	0.791	—	0.823	1.158	2.398	—
GCN [2017]	0.877	0.854	0.740	0.807	1.068	2.900	—
MPNN [2017]	0.913	0.815	0.770	0.879	1.167	2.185	111.4
DMPNN [2019]	0.919	0.852	0.771	0.897	0.980	2.177	103.5
CMPNN [2020]	0.927	0.869	0.782	0.902	0.798	0.956	75.1
CoMPT [2021]	0.938	0.871	—	**0.934**	0.774	1.855	65.3
FP-GNN [2022]	0.916	0.860	0.824	0.840	0.675	0.905	—
SG-ATT	**0.943**	**0.910**	**0.827**	0.920	**0.646**	**0.879**	**64.1**

Bold cells indicate the best results achieved, underlined cells indicate the second-best results achieved, and “—” indicates that the model did not undergo corresponding experimental testing.

**Table 2:** Results of SG-ATT ablation experiments.

Metric	ROC-AUC				RMSE		MAE
Dataset	BBBP	BACE	HIV	ClinxTox	ESOL	FreeSolv	QM7
SG-ATT	**0.943**	**0.910**	**0.827**	**0.920**	**0.646**	**0.879**	**64.1**
-w/o Graph	0.883	0.864	0.757	0.853	0.858	1.126	68.7
-w/o Sequnce	0.922	0.879	0.788	0.910	0.692	0.904	67.3

Bold cells indicate the best results achieved.

**Table 3:** Test results on CHEMBL screening data and in vitro validation data.

Dataset	ROC-AUC
CHEMBL_CC50_	0.817
in vitro validation_CC50_	0.704
CHEMBL_IC50_	0.783
in vitro validation_IC50_	0.696

**Table 4:** Top 10 compounds for predicting IC_50_.

Rank	SMILES	In Vitro Validation (μM)
1	O=C(N[C@H](CO)CC1=CC=CC=C1)/C(NC(C2=C(F) C=CC=C2)=O)=C\C3=CC=CC=C3	>20
2	O=C(N[C@H](CO)CC1=CC=CC=C1)/C(NC(C2=CC=CC=C2)=O) =C\C3=CC=CC=C3Br	>20
3	O=C(N[C@H](CO)CC1=CC=CC=C1)/C(NC(C2=CC=CC=C2)=O) =C\C3=CC=CC=C3Cl	>20
4	Br/C(C1=CC=CC=C1)=C(NC(C2=CC=CC=C2)=O)/ C(N[C@H](CO)CC3=CC=CC=C3)=O	3.86
5	Br/C(C1=CC=C(Br)C=C1)=C(NC(C2=CC=CC=C2)=O)/ C(N[C@@H](CC3=CC=CC=C3)CO)=O	4.45
6	O=C(N[C@H](CO)CC1=CC=CC=C1)/C(NC(C2=CC=CC=C2) =O)=C\C3=CC=CC(C)=C3	>20
7	O=C(N1CCCCC1)[C@@H](NC(C2=CC=CC=C2) =O)CC3=CC=CC=C3	>20
8	Br/C(C1=CC=CC=C1OC)=C(NC(C2=CC=C([N+]([O-]) =O)C=C2)=O)/C(N[C@@H](CO)C(C)C)=O	2.06
9	Br/C(C1=CC=CC=C1Cl)=C(NC(C2=CC=CC=C2)=O)/ C(N[C@@H](CC3=CC=CC=C3)CO)=O	1.88
10	O=C1C2=CC(OCCN(CC)CC)=CC=C2N=C (SCC3=C(OC)C=CC=C3)N1CC4=CC=CS4	1.93

**Table 5:** Top 10 compounds for predicting CC_50_.

Rank	SMILES	In Vitro Validation (μM)
1	Br/C(C1=CC=C(O)C=C1)=C(NC(C2=CC=CC=C2)=O)/ C(N[C@H](CO)CC3=CC=CC=C3)=O	>100
2	O=C(N[C@H](CO)CC1=CC=CC=C1)/C(NC(C2=C(F) C=CC=C2)=O)=C\C3=CC=CC=C3	>100
3	O=C(N[C@H](CO)CC1=CC=CC=C1)/C(NC (C2=CC=CC=C2)=O)=C\C3=CC=CC=C3Cl	>100
4	Br/C(C1=CC=CC=C1Br)=C(NC(C2=CC=CC=C2)=O)/ C(N[C@@H](CC3=CC=CC=C3)CO)=O	>100
5	O=C(N[C@H](CO)CC1=CC=CC=C1)/ C(NC(C2=CC=CC=C2)=O)=C\C3=CC=CC(C)=C3	>100
6	O=C(N[C@H](CO)CC1=CC=CC=C1)/C (NC(C2=CC=CC=C2)=O)=C\C3=CC=C(Br)C=C3	72.66
7	Br/C(C1=CC=C(C#N)C=C1)=C(NC(C2=CC=CC=C2)=O)/ C(N[C@@H](CC3=CC=CC=C3)CO)=O	>100
8	O=C(N/C(C(N1CCCCC1)=O)=C(Br)/C2=NC=CS2) C(C=C3)=CC=C3F	>100
9	O=C(NC(CO)C(C)C)[C@@H](NC(C1=CC=CC=C1)=O)CC2=CC=C(OCCN(C)C)C=C2	89.10
10	O=C(N[C@H](CC(C)C)CO)/C(NC(C1=CC=C([N+]([O-])=O) C=C1)=O)=C(Br)\C2=CC=CC=C2OC	86.61

## Data Availability

All datasets and codes used in this study are available at GitHub: https://github.com/NTU-MedAI/SG-ATT (accessed on 20 April 2023).

## Appendix A. Details of AMPNN

KMPNN builds upon MPNN by considering the establishment of transmission channels for different types of messages, utilizing two distinct message-passing channels. AMPNN, based on KMPNN, considers the creation of distinct message aggregation methods for the transmission of different message types, enabling a more diverse aggregation of various message types. Figure A1 illustrates the architecture of AMPNN. It represents different types of nodes in the molecular graph, including atoms and attributes, through heterogeneous message passing. During the message delivery process, nodes of the same type share message parameters, and different parameters are used to calculate attention coefficients between atoms and attributes, as well as between atoms and other atoms. Additionally, in the message aggregation stage, different types of messages are assigned different feature aggregation methods.

## Appendix B. Details of GRU and Transformer

The Gated Recurrent Unit (GRU) is proposed to address the problems of long-term memory and gradient in backpropagation. The GRU combines the current input $x^{t}$ with the hidden state $h^{t-1}$, passed from the previous node (this hidden state contains the relevant information of the previous node), and the GRU produces the output $y^{t}$ of the currently hidden node and the hidden state $h^{t}$ passed to the next node.

The encoder of the Transformer encodes an input sequence of symbol representations x= ($x_{1}$, …, $x_{n}$) to a sequence of continuous representations z= ($z_{1}$, …, $z_{n}$). Given *z*, the decoder then generates an output sequence y= ($y_{1}$, …, $y_{n}$) of symbols one element at a time. At each step, the model is auto-regressive, consuming the previously generated symbols as additional input when generating the next. An attention function can be described as mapping a query and a set of key-value pairs to an output, where the query, keys, values, and output are all vectors. The output is computed as a weighted sum of the values, where the weight assigned to each value is computed using a compatibility function of the query with the corresponding key. The Transformer follows this overall architecture using stacked self-attention and pointwise, fully connected layers for both the encoder and decoder.

## Appendix C. Details of Dataset

### Appendix C.1. Dataset Description

Table A1 summarizes the information on the benchmark datasets, including the task type, dataset size, and split type. Detailed information for each dataset is shown below. These datasets play a significant role in the fields of chemistry and pharmaceutical research, holding practical value for tasks such as developing predictive models and conducting drug design.

**Table A1:** Dataset information.

Type	Category	Dataset	Tasks	Compounds	Split
Classification	Biophysics	BACE	1	1513	Scaffold
		HIV	1	41,127	Scaffold
	Physiology	SIDER	27	1427	Random
		ClinTox	2	1478	Random
		BBBP	1	2039	Scaffold
		Tox21	12	7831	Random
		ToxCast	617	8575	Random
Regression	Physical chemistry	FreeSolv	1	642	Random
		ESOL	1	1128	Random
	Quantum mechanics	QM7	1	7160	Random

The BACE dataset consists of drug molecules with diverse structural characteristics, serving as a platform for predicting bioactivity against beta-secretase [42].

The HIV dataset includes compounds along with their viral inhibition activities, providing crucial insights for antiretroviral drug research [43].

The BBBP dataset evaluates blood–brain barrier permeability prediction using molecular features, aiding in an understanding molecule–brain interactions [44].

The ClinTox dataset encompasses drug compounds and associated toxicity labels, offering valuable insights into drug safety assessment [45].

The Tox21 dataset provides molecular features and covers diverse toxicity activity labels, facilitating toxicity prediction tasks [46].

The ToxCast dataset features various toxicity activity data, offering a comprehensive resource for predictive toxicology [47].

The SIDER dataset includes molecular structures and drug side effect labels, facilitating the analysis of drug safety profiles [48].

The ESOL dataset focuses on predicting the solubility of different molecules, contributing to drug formulation studies [49].

The FreeSolv dataset involves predicting the free energy changes of diverse molecular complexes, a valuable resource for free energy calculations of solvation [50].

QM7: This is a dataset designed for quantum chemistry calculations aimed at studying the electronic structures of small organic molecules and predicting the quantum mechanical properties of molecules [51].

### Appendix C.2. Dataset Splitting

As shown in Table A1, molecular skeleton-based splitting and random splitting were applied to all tasks for all datasets [52]. Molecular skeleton-based splitting splits molecules with different 2D structural frameworks into different subsets. This is a more challenging but practical setup because the test molecules may have different structures from the training set. All datasets were divided into training, validation, and test sets at a ratio of 8:1:1. In addition, scaffold splitting was performed for the BACE, BBBP, and HIV datasets, and random splitting was used for the remaining datasets.

## Appendix D. Specific SMILES for Each Molecular Graph

Table A2 lists the specific SMILES representations corresponding to each molecule.

**Table A2:** Specific SMILES.

Name	SMILES
SMILES.1	CC(=O)Nc1ccccc1
SMILES.2	O=C(C)Nc1ccccc1C
SMILES.3	CCC(C)=O
SMILES.4	CC(C)CO
SMILES.5	Cc1ccccc1C
SMILES.6	Cc1ccccc1
SMILES.7	CCOCC(=O)Nc1ccccc1C
SMILES.8	O=C(C)OC(=O)c1ccccc1C
